# Endoscopic management of bilateral frontal mucopyoceles: A Case Report and Literature review

**DOI:** 10.1016/j.ijscr.2020.02.039

**Published:** 2020-02-26

**Authors:** Zainab Alhassan, Fadel Molani, Ali Almomen

**Affiliations:** a Department of Medical Imaging, KFSH, Dammam, Saudi Arabia; b Department of ENT, KFSH, Dammam, Saudi Arabia; c King Fahad Specialist Hospital, Dammam, Saudi Arabia

**Keywords:** KFSH, King Fahad specialist hospital, FESS, functional endoscopic sinus surgery, EMLP, endoscopic modified Lothrop procedure, OPF, osteoplastic flap surgery, Frontal sinus, Bilateral, Mucocele, Endoscopic, Image guided, FESS

## Abstract

**Objective:**

To demonstrate the effectiveness of the endonasal endoscopic approach in managing a large bilateral frontal mucopyocele causing a mass effect on the brain.

**Case presentation:**

A 17 year old chronic sinusitis patient, presented with bilateral nasal obstruction and increasing left sided headache; was found to have a very large bilateral frontal mucopyoceles with extensive intracranial extradural brain involvement on CT scan and MRI of the sinuses and brain which was drained and evacuated completely using the endonasal endoscopic image guided approach with no complications.

**Conclusion:**

The endonasal endoscopic image guided approach is the approach of choice in managing large frontal mucopyocele with brain extension.

It is a safe, accurate, reliable approach with no complications.

## Highlights

• Surgery is the modality of choice to achieve complete evacuation of the mucocele and reestablishing the normal sinus outflow.
• In determining the surgical approach to mucoceles, two main factors are considered: the patency of the ipsilateral frontal sinus outflow and the accessibility of the sinus via endoscope.
• The endonasal endoscopic image guided approach is a safe, accurate, and reliable approach with no complications.
• The concept behind the utility of image guidance in endoscopic marsupialization.
• It is the approach of choice in managing large frontal mucopyocele with brain extension.

## 1 Introduction

Mucocele is a benign, mucosa lined pseudocystic lesions of the paranasal sinus that, due to mucus secretion, has a tendency to expand giving rise to mass effect on surrounding structures. This may result in erosion and reabsorption of the bony sinus walls. They develop due to obstruction of the sinus ostium by congenital anomalies or acquired causes such as allergic rhinitis, post-traumatic, post-inflammation and infection [1]. The commonly affected is the frontal sinuses (60 to 89%) whereas ethmoidal sinuses are less common (8 to 30%) with maxillary sinus being the least frequent (<5%) [1,2].

Mucoceles may presents with variable manifestations that are based on the location of mucocele and its extent. Symptoms might be rhinologic, neurologic or ophthalmologic [2].

CT scan is the imaging modality of choice with MRI being superior in terms of differentiating a mucocele from soft tissue tumors as well as localizing the mucocele in relation to brain and orbit [3].

We hereby report a rare case of bilateral frontal mucopyoceles managed by image guided endoscopic drainage and marseuplization at our hospital in Saudi Arabia.

This case report has been reported in line according to Surgical Case Reports (SCARE) Criteria [4].

## 2 Case report

A 17-year-old male patient presented to ENT clinic with history of facial heaviness and chronic headache located in the left frontal area. Associated with bilateral nasal obstruction, anosmia and recurrent epistaxis. His medical history includes diabetes mellitus type 2 on insulin regimen. His surgical history includes FESS done one year back. Ophthalmic examination showed displaced left eye downward and outward. ENT examination showed bilateral nasal pale polyps grade 3-4 with white discharge. Otherwise examination was unremarkable.

**Figs. 1–3:**
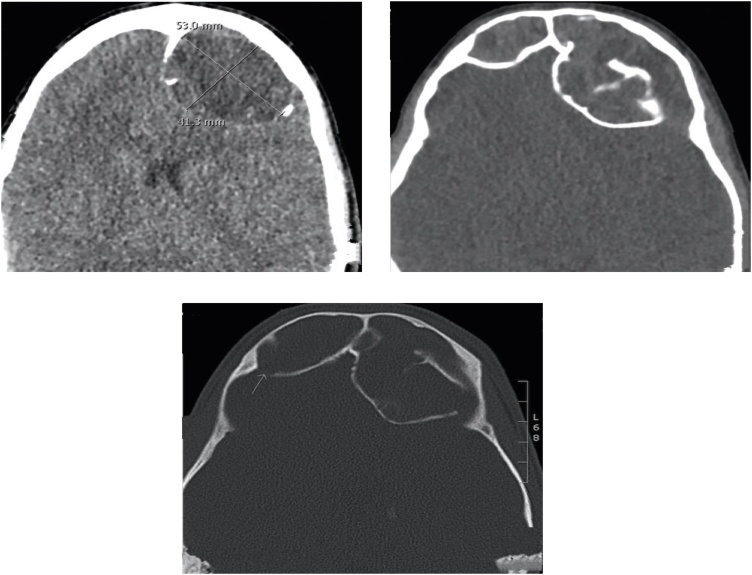
Marked enlargement of the frontal sinuses, more pronounced on the left side with associated dehiscence of the posterior wall of the sinus.

The patient was evaluated by CT scan of the brain and paranasal sinuses which revealed a marked enlargement of the frontal sinuses, more pronounced on the left side with associated dehiscence of the posterior wall of the sinus and intracranial extension of soft tissues within epidural space measuring 5.3 × 4.1 cm on axial image (Figs. 1–3).

**Figs. 4–6:**
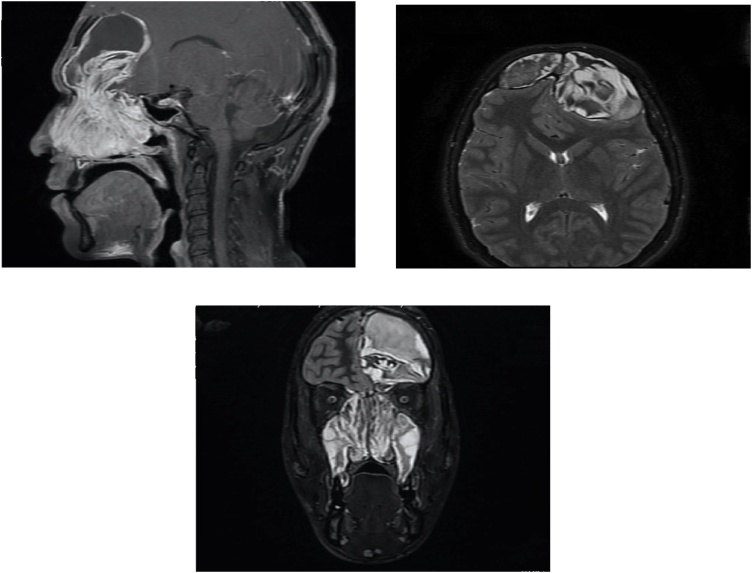
MRI of the brain and sinuses showed significant expansion of both frontal sinuses by the mucopyoceles.

MRI of the brain and sinuses (Figs. 4–6) showed significant expansion of both frontal sinuses by the mucopyoceles with largest on the left measuring 4.7 × 6.5 × 6.7 cm with avid heterogeneous enhancement. The mucopyoceles were displacing and compressing the underlying dura with significant mass effect (Figs. 4 and 5).

**Fig. 7:**
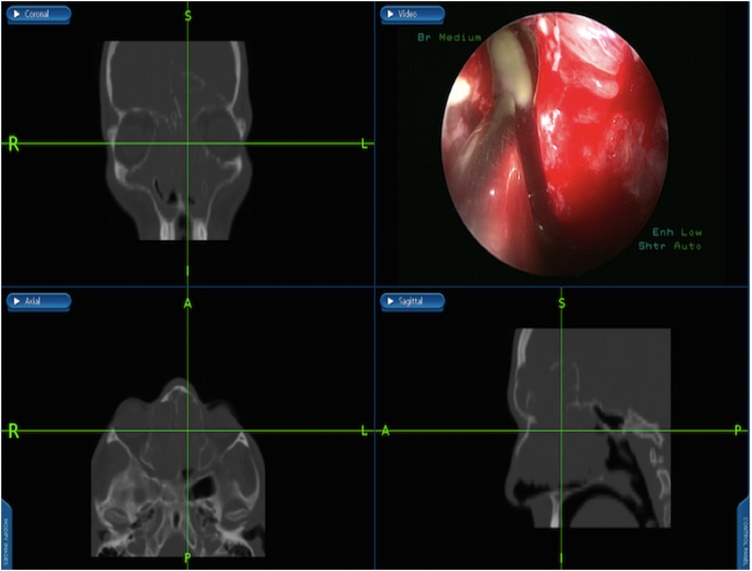
Frontal recess identified showing obstructing infected polyps that was removed and drained by image-guided endoscopic sinus surgery.

**Fig. 8:**
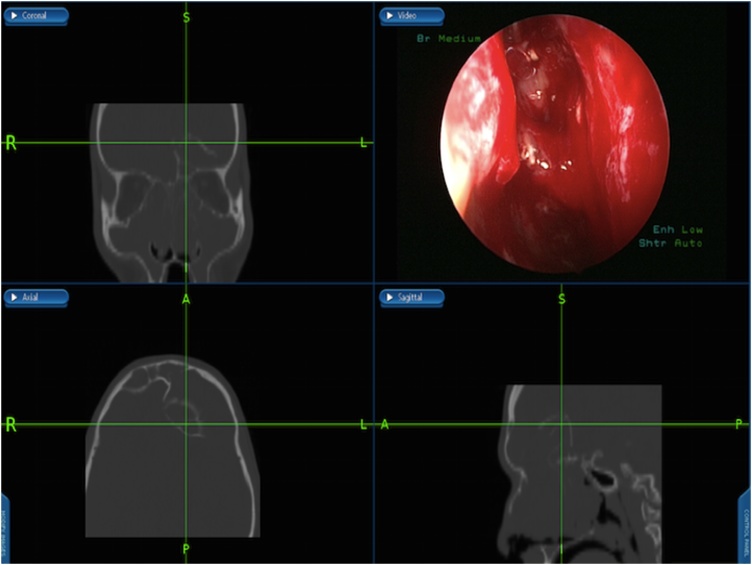
Draf type 2b performed, mucopyoceles cavities full of pus irrigated with antibiotic soaked irrigations.

**Fig. 9:**
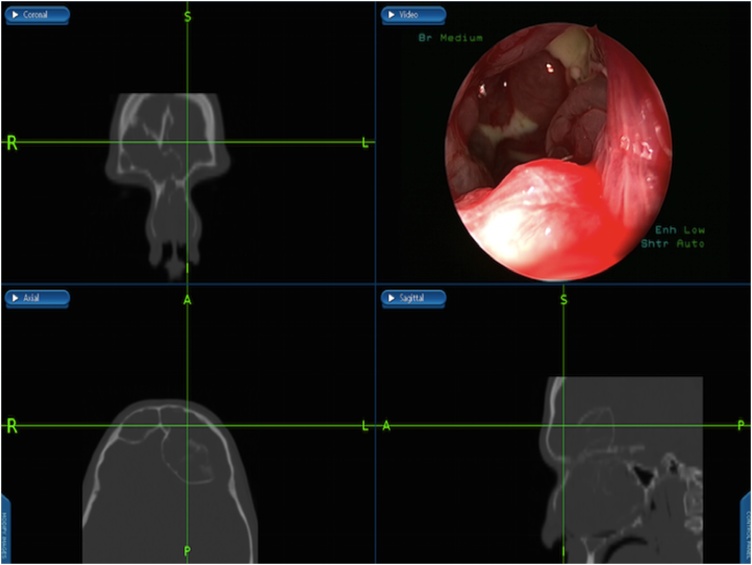
Both mucopyoceles cavities were evacuated.

Under general anesthesia, image guided endoscopic sinus surgery was undertaken. Examination revealed extensive nasal polyposis grade 4 that was removed by microdebrider and frank pus was drained, removal of the obstructing polyps from the ethmoidal cavities was done, frontal recess was identified full of obstructing infected polyps that was removed and drained (Fig. 7). Draf type 2 b procedure was performed bilaterally to adequately drain and ventilate the frontal sinuses (Fig. 8), that facilitate identifying the mucopyoceles cavities which were full of frank mucopus that was drained and the cavities were irrigated with antibiotic soaked irrigations (Fig. 8). The cavities of both mucopyoceles were completely evacuated and checked by the help of navigation (Fig. 9).

**Fig. 10:**
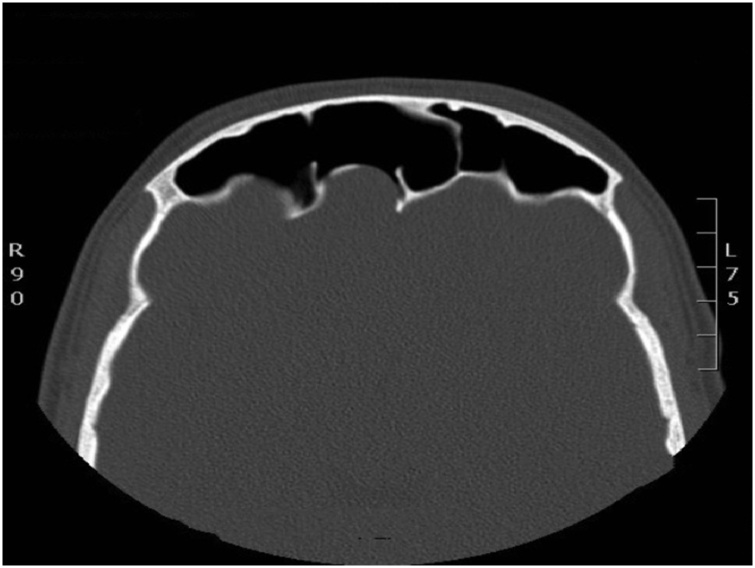
Post op Axial CT sinus.

**Fig. 11:**
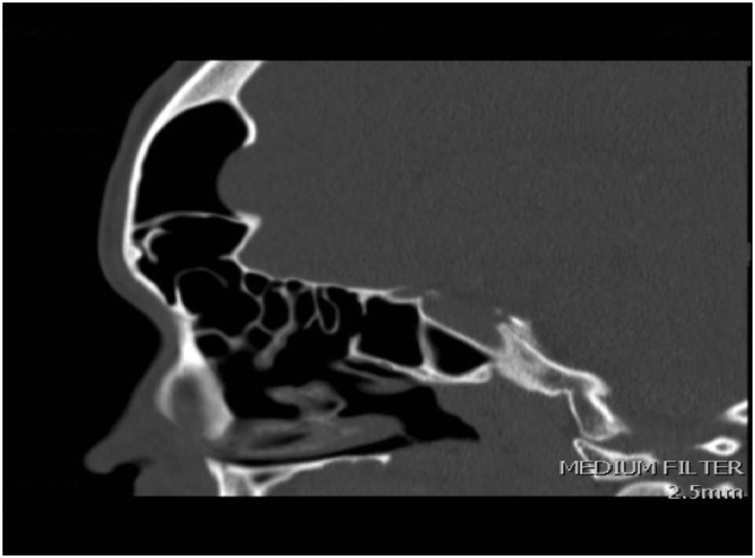
Post op Sagittal CT sinus.

6 months post operative follow up CT scan of the sinuses showed complete resolution and normal aeration of the sinuses (Fig. 10, Fig. 11)

## 3 Discussion

Mucoceles are slowly growing, mucus containing lesions that develops in the paranasal sinuses. A chronic expanding mucocele with mucous retention can become infected causing mucopyocele [2]. There are a limited number of cases in the literature, which reported bilateral frontal mucocele, nonetheless, frontal sinus mucocele is common, only seven cases reported bilateral frontal involvement [5].

Frontal mucocele may presents with a variable spectrum of symptoms based on the location and extent of the mucocele; symptoms may include nasal obstruction or postnasal drip, headache, dizziness, maxillofacial pressure, subcutaneous forehead or periorbital tenderness or swelling. If the mucocele has invaded the orbital cavity, patients may experience diplopia, ptosis, proptosis and globe displacement [2,3,6].

Surgery is the modality of choice to achieve complete evacuation of the mucocele and reestablishing the normal sinus outflow [7]. In the last decade, with the development in surgical techniques and usage of image guidance, the management of paranasal sinuses has improved. Despite this fact, the choice of a certain surgical approach to a given mucocele localization has not yet been universally accepted. The ultimate approach should provide a definitive resolution without recurrence and maintain the normal drainage architecture [6].

For fronto-ethmoidal mucoceles in particular, there were many controversies in previous literature regarding recurrence rates, surgical morbidity and preservation of normal sinus outflow [6].

Before the availability of intraoperative image guidance in paranasal surgeries, open and combined surgical approaches were undertaken for frontal lateral mucoceles where they caused extensive scarring. With the advances in technology and the availability of equipment and adequate training, endoscopic marsupialization with image guidance could become the surgical choice for managing complex mucoceles [[8], [9], [10]].

The concept behind image guidance navigation system is using an infrared camera or radiofrequency to track along the surgical instrument in relation to the patient’s head during operation. A computer set will process this information and delineate the tip of the instrument over the patient’s preoperative sinus CT scan by a 3-dimensional video display. This technology allows the surgeon to confirm the anatomy of the sinus and its location relative to adjacent structures such as skull and orbits [11].

Surgical approach to treat mucuceles is challenging due to many factors, including complex and variable anatomy, possible scarring and stenosis. In determining the surgical approach to mucoceles, two main factors are considered: the patency of the ipsilateral frontal sinus outflow and the accessibility of the sinus via endoscope. When these factors are not met, a more invasive external approach is undertaken such as endoscopic modified Lothrop procedure (EMLP) or an osteoplastic flap surgery (OPF) with or without frontal sinus obliteration [[8], [9]]. The EMLP has many advantages over OPF surgery; nevertheless, it is a difficult surgical technique and has major complications such as CSF leak [8].

Recently, endoscopic approach is the mainstay of management for paranasal sinus mucoceles with lower rates of morbidity [[7], [8], [9], [10], [11], [12]]. In the case presented here, an endonasal endoscopic approach was performed with CT guided navigation system for safe drainage, accurate localization, complete evacuation and marseuplization of the mucopyoceles cavities.

Endonasal endoscopic mucocele management has the advantage of allowing a less traumatic approach, as well as reducing morbidity rates and operative time to a minimum. Therefore, endoscopic surgery is becoming the surgical technique of choice [2,8].

Several possible complications can arise due to the close proximity of the paranasal sinuses and the orbital cavity; Inattentive direct trauma to orbital content during surgery is potential. Susceptibility of direct damage or entrapment of the medial rectus near the lamina papyracea; direct damage to optic nerve and intraorbital hemorrhage are all possible [2,13]. Postoperative complications may also occur, including intraorbital hematoma, abscesses, or mucocele recurrence [2,7]. With the expanding mucocele, it can erode the posterior wall of the frontal sinus leading to complications including epidural abscess, meningitis, subdural empyema and possibly brain abscess [1,2,8,13].

## 4 Conclusion

The endonasal endoscopic approach to large frontal mucopyoceles is successful, with low morbidity and low recurrence rates.

The image guided Endoscopic drainage and marsupialization is a safe, reliable and accurate in managing large frontal mucopyocele with brain involvements. This approach, when compared with open techniques, is associated with low recurrence rates and low rates of complications.

## Sources of funding

None.

## Ethical approval

The ethical approval is exempted for the case report at our institution.

## Consent

Written informed consent was obtained from the parents for publication of this case report and images provided on behalf of the patient.

## Author contribution

**Zainab Alhassan:** Manuscript draft and final edits.

**Fadel Molani:** Data collection and literature review.

**Ali Almomen:** Operating surgeon, data analysis and interpretation and critical revision of the manuscript.

## Registration of research studies

Not Applicable.

## Guarantor

Ali Almomen.

## Patient counsel

Written informed consent was obtained from the parents for publication of this case report on behalf of the patient.

## Data availability

The data used to support the findings of this study are included within the article. Also, they are available from the corresponding author upon request.

## Methods

This work has been reported in line with the SCARE criteria.

## Provenance and peer review

Not commissioned, externally peer-reviewed.

## Declaration of Competing Interest

The authors declare that there is no conflict of interest regarding the publication of this paper.
